# pH-Sensitive Chitosan–Heparin Nanoparticles for Effective Delivery of Genetic Drugs into Epithelial Cells

**DOI:** 10.3390/pharmaceutics11070317

**Published:** 2019-07-05

**Authors:** Iuliia Pilipenko, Viktor Korzhikov-Vlakh, Vladimir Sharoyko, Nan Zhang, Monika Schäfer-Korting, Eckart Rühl, Christian Zoschke, Tatiana Tennikova

**Affiliations:** 1 Institute of Chemistry, Saint Petersburg State University, Peterhoff, Universitetskii pr. 26, 198504 St. Petersburg, Russia; 2 Institute of Pharmacy (Pharmacology and Toxicology), Freie Universität Berlin, Königin-Luise-Straße 2+4, 14195 Berlin, Germany; 3 Institute of Chemistry and Biochemistry (Physical Chemistry), Freie Universität Berlin, Arnimallee 22, 14195 Berlin, Germany

**Keywords:** chitosan, cytotoxicity, DNA, heparin, transfection, pH-sensitive

## Abstract

Chitosan has been extensively studied as a genetic drug delivery platform. However, its efficiency is limited by the strength of DNA and RNA binding. Expecting a reduced binding strength of cargo with chitosan, we proposed including heparin as a competing polyanion in the polyplexes. We developed chitosan–heparin nanoparticles by a one-step process for the local delivery of oligonucleotides. The size of the polyplexes was dependent on the mass ratio of polycation to polyanion. The mechanism of oligonucleotide release was pH-dependent and associated with polyplex swelling and collapse of the polysaccharide network. Inclusion of heparin enhanced the oligonucleotide release from the chitosan-based polyplexes. Furthermore, heparin reduced the toxicity of polyplexes in the cultured cells. The cell uptake of chitosan–heparin polyplexes was equal to that of chitosan polyplexes, but heparin increased the transfection efficiency of the polyplexes two-fold. The application of chitosan–heparin small interfering RNA (siRNA) targeted to vascular endothelial growth factor (VEGF) silencing of ARPE-19 cells was 25% higher. Overall, chitosan–heparin polyplexes showed a significant improvement of gene release inside the cells, transfection, and gene silencing efficiency in vitro, suggesting that this fundamental strategy can further improve the transfection efficiency with application of non-viral vectors.

## 1. Introduction

Progress in the field of gene medicine has been stimulated by basic research of novel vectors for DNA, RNA, and oligonucleotides. The use of different nucleotide-based therapeutics (e.g., siRNA, messenger RNA (mRNA), and plasmid DNA (pDNA)) is an important strategy in future treatment of diseases [1]. The cellular mechanisms of listed therapeutic agents are extremely different, with structural advantages [2]. Nevertheless, it is important to find an effective delivery system for genetic medicines to obtain high therapeutic efficiency in the cell.

Cationic biopolymers have been frequently used as a nano-carriers for siRNA and pDNA delivery because they effectively complex the anionic polynucleotides, protect them from degradation and improve their cellular uptake [3]. The colloidal vesicles of hydrophilic natural polymers, such as chitosan and its derivatives, represent a promising class of such biopolymers [4,5,6]. Chitosan is water-soluble and positively charged polymer with pK_a_ of 6.0. It forms pH-sensitive nano-sized hydrogels that may enable pH-triggered release siRNA or pDNA in the acidic medium of endosomes [7,8]. The positive charges of chitosan can be used to form colloidal nanoparticles via electrostatic interactions with polyanions and to promote their internalization into the cells. Moreover, chitosan adheres to mucosal surfaces [9] and enhances the epithelial penetration by opening tight-junctions, which is key to overcoming the skin barrier [10]. In addition, chitosan is metabolized by certain human enzymes [11] and it is considered a biocompatible polymer with low toxicity [12].

Numerous excellent reviews and regular articles on the potential of chitosan for pharmaceutical applications have been published recently [13,14,15,16]. Despite the great potential of chitosan [13], its efficiency for delivering different types of oligo- and polynucleotides strongly depends on the N/P (amino groups of polycation to phosphate groups of polyanion) ratio, molecular structure, size, and shape of the complexes. High N/P ratios in the complexes lead to high stability of the polyplexes that prevents release of the cargo within the cells and precludes translation of DNA or RNA action [15,16]. To address this issue, we incorporated heparin as a stronger polyanionic competitor for polynucleotides.

Alternative polyplexes with other polyanions, such as sodium alginate [17] and hyaluronic acid [18], have already been published as tools for improved gene delivery into epithelial cells. Sodium alginate and hyaluronic acid have a gelling behavior, high molecular weight, and broad molecular weight distribution, which can lead to aggregation and reduced stability of the particles. Heparin is biocompatible and mucoadhesive biopolymer with comparatively low molecular mass (12–14 kDa), non-gelling behavior, and stronger acidic sulfate groups. Thus, we propose that heparin could be more effective competitive polyanion for improved RNA or DNA release from the polyplexes.

The formulations based on chitosan–heparin nanoparticles were previously described [19,20]. In one study [19], chitosan–heparin particles were used for bovine serum albumin (BSA) delivery. The physico-chemical properties of such formulations, namely, size, pH-sensitivity, zeta-potential, and entrapment efficiency of BSA were described. In another study, the immobilization of VEGF-loaded chitosan–heparin nanoparticles on the scaffolds for tissue-engineering was performed [20]. This significantly increased fibroblast infiltration, extracellular matrix production, and accelerated in vivo vascularization in a mouse subcutaneous implantation model. Thus, the application of chitosan–heparin carriers as a highly biocompatible and effective delivery system represents a promising approach for genetic drug delivery into the cells.

In this work we developed pH-sensitive chitosan–heparin nanoparticles and investigated the effect of different chitosan/heparin ratios on pH, particle size and structure, zeta potential, and encapsulation efficiency of model oligonucleotide. Next, we studied the release mechanisms of model oligonucleotide and proved the biocompatibility of the drug delivery system with primary normal human keratinocytes (NHKs). Finally, we unraveled specific role of competitive polyanion heparin in the complex and showed the advances of chitosan–heparin nanocarrier to deliver plasmid DNA and VEGF silencing RNA to epithelial cells.

## 2. Materials and Methods

### 2.1. Materials

Polymers for nanoparticle preparation include chitosan (deacetylation degree 75–85%, medium molecular weight; Sigma Aldrich, Schnelldorf, Germany) and heparin (MW 12–14 kDa; AppliChem, Darmstadt, Germany). Model Cy3-labeled and non-labeled oligothymidine and oligoadenine (23 base pairs oligo-dT-dA) were purchased from Biobeagle^TM^ (Saint-Petersburg, Russia).

The reporter gene plasmid encoding green fluorescent protein (pEGFP-C2) was a generous gift from Prof. Arto Urtti (University of Eastern Finland, Kuopio, Finland). pEGFP-C2 was transformed into *Escherichia coli* XL1-Blue (Evrogen, Moscow, Russia) and purified using the Plasmid Miniprep purification kit (Evrogen, Moscow, Russia). DNA concentration was quantified by the measurement of UV absorbance at 260 nm using a Nanodrop 2000c spectrophotometer (Thermo Fischer Scientific, Vantaa, Finland). The purity of the plasmid was verified by gel electrophoresis (1% agarose gel).

The 27-base pairs (bp) double stranded (ds) RNA (sense and antisense strands) were designed as targets to the vascular endothelial growth factor gene (sense: 5′-CUUCCUACAGCACAACAAAUGUGAAUG-3′, antisense: 3′GAAGGAUGUCGUGUUGUUUACACUUAC-5′). For visualization, Cy5-labeled (5′-modification) 27-bp VEGF siRNA were used. Cy5-labeled and non-labeled 27-bp ds VEGF siRNAs and scrambled 27-bp RNA (siC) (sense 5′-GUAAGUGUAAACAACACGACAUCCUUC-3′, antisense: 3′-CAUUCACAUUUGUUGUGCUGUAGGAAG-5′ [21] were purchased from GenTerra (Moscow, Russia). The primers used for the target mRNA: VEGF forward primer (5′-CCCTGATGAGATCGAGTACATCTT-3′), VEGF reverse primer (5′-ACCGCCTCGGCTTGTCAC-3′), GAPDH forward primer (5′-GTCTCCTCTGACTTCAACAGCG-3′), and GAPDH reverse primer (5′-ACCACCCTGTTGCTGTAGCCAA-3′) were purchased from GenTerra (Moscow, Russia).

Primary NHKs were from therapeutically indicated circumcisions (ethical approval EA1/081/13) after parents had signed the written informed consent. Primary cells were isolated according to a standard operating procedure [22]. In brief, tissues were washed with phosphate buffered saline (PBS) pH 7.4, cut into pieces, and incubated with dispase solution overnight at 4 °C. Next, epidermis was separated from dermal pieces and collected in Trypsin–EDTA solution to isolate keratinocytes. After stopping the enzymatic reaction, keratinocytes were grown and subcultured in cell culture flasks. Cells in passage 3 were used for cell viability experiments.

Human retinal epithelial (ARPE-19) cells were obtained from American Type Culture Collection (ATCC, Manassas, VA, USA).

### 2.2. Particle Preparation

Chitosan–heparin nanoparticles were prepared based on spontaneous polyelectrolyte complexation in mild conditions [15]. Polyelectrolyte complex formation between polycationic chitosan and polyanionic heparin results in partial charge neutralization. Before complexation heparin was dissolved in deionized water to give solution with concentration 1 mg/mL, while chitosan was dissolved in 0.01% acetic acid solution to obtain 10 mg/mL solution and further diluted with PBS pH 8.0–8.5 or 0.1M NaOH to obtain the final concentration 0.1 mg/mL. The pH of the chitosan solutions was fixed at 6.0, except in the studies on the effects of pH on size/zeta-potential and encapsulation efficiency of oligonucleotide. Before the complexation, chitosan solution was sonicated for 60–90 s using 10% power of ultrasonic homogenizer (Bandelin Sonopuls HD 2070, Berlin, Germany). After that, chitosan–heparin nanoparticles were obtained by dropwise addition of heparin solution to chitosan solution under stirring (Vortex, Thermo Fischer Scientific, Vantaa, Finland) at room temperature. To encapsulate oligo- and polynucleotides inside chitosan–heparin nanoparticles, the anionic oligonucleotide was added to chitosan solution immediately after sonication under stirring, thereafter heparin was added to these complexes.

### 2.3. Particle Structure

Hydrodynamic diameter, polydispersity index (PDI) and zeta potential were determined by dynamic light scattering (DLS) using a Zetasizer Nano ZS with He-Ne laser (λ = 633 nm; Malvern Instruments, Worcester, UK) at 25 °C. Moreover, particle morphology and size were measured with scanning-transmission electron microscopy (STEM; SU8010, Hitachi, Japan) and nanoparticle tracking analysis (NTA; Nanosight NS300, Malvern Panalytical, Worcester, UK).

The concentration of particles which were used for DLS, zeta-potential, and STEM experiments was 10% from the stock particle suspension. DLS experiments were performed in PBS, pH 7.4, whereas the zeta-potentials were determined in deionized water (except the studies of pH effects).

### 2.4. Oligonucleotide Loading Efficiency

Oligonucleotide-loaded nanoparticles were obtained by adding 1 μg of duplex oligo-thymidine-adenine (dT-dA) or Cy3-oligo-thymidine-adenine (Cy3-dT-dA) to chitosan solution before complexation with heparin. To determine the loading efficiency of Cy3-dT-dA into chitosan–heparin nanoparticles, free oligonucleotide was separated from the loaded nanoparticles using centrifugation of 10,000× *g* at 4 °C for 20 min in filter tubes (30,000 NWML, Amicon Ultra 0.5 mL, Merck, Darmstadt, Germany). The filtrate with free Cy3-dT-dA was collected and analyzed using multimode microplate reader (Thermo Scientific Varioscan lux, Vantaa, Finland) at excitation and emission wavelengths of 550 and 570 nm, respectively. The amount of loaded oligonucleotide was calculated using a linear calibration curve. The loading efficiency was expressed as encapsulated/total ratio of oligonucleotide.

### 2.5. Oligonucleotide Release

In vitro release of Cy3-dT-dA from chitosan–heparin and chitosan complexes was measured for 4 h using 100 μL of test formulation diluted with 300 μL of the release media. The release media were buffer solutions with different pH: late endosome (2-(*N*-morpholino)ethanesulfonic acid buffer solution (MES) pH 6.3), lysosome (MES pH 4.5), cytosol (PBS pH 7.4) media, in Eppendorf tubes with filter (30,000 NWML, Amicon Ultra 0.5 mL, Merck, Darmstadt Germany) and shaken at 37 °C and 1000 rpm. At predetermined times, the tubes were centrifuged at 10,000× *g* for 15 min. The filtrates were collected for fluorescent measurements to estimate the amount of released oligonucleotide (λ_ex_ = 550 nm, λ_em_ = 570 nm). The release was calculated as slope × fluorescence × volume of sample.

### 2.6. Particle Cytotoxicity

The cytotoxicity of nanoparticles was studied using MTT (3-(4,5-dimethylthiazol-2-yl)-2,5-diphenyltetrazolium bromide) reduction assay. NHK were seeded into 96-well plates at density of 10^4^ cells per well (TPP, Trasadingen, Switzerland). Keratinocyte growth medium (KGM) was prepared by mixing of keratinocyte basal medium (KBM, Lonza, Visp, Switzerland) with a KGM supplement pack, which contained 2.0 mL of BPE, 0.5 mL human epidermal growth factor protein (hEGFP), 0.5 mL of insulin, 0.5 mL of hydrocortisone, 0.5 mL of transferrin, 0.5 mL of epinephrine, and 0.5 mL of GA-1000. After 24 h the nanoparticles without any cargo and reagents for particle preparation (concentrations of 0.05% and 0.005% mass referred to chitosan amount in KGM) were added for a 24-h period. Then, 100 μL MTT solution (5 mg/mL in PBS) was added after the incubation period for 4 h. The supernatant was carefully removed, and cells were lysed in 50 μL dimethyl sulfoxide for 5 min to dissolve the formazan salt crystals. The absorbance was measured at 540 nm using a Micro Plate Reader (FLUOstar Optima, BMG Labtech, Ortenberg, Germany). For toxicity assessment, KGM was used as a reference for untreated cells (negative control), 0.005% mass. Sodium dodecyl sulfate (SDS, Sigma-Aldrich, München, Germany) in KGM was used as positive control, and 10% of distilled water in KGM was the solvent control. The mean value of solvent control (corrected for blank value) was set to 100%. As reported in the previous studies, we considered cell viability below 75% as an indicator of cytotoxicity [23].

In addition to the testing of NAD(P)H-dependent oxidoreductase activity using MTT assay, the Trypan Blue dye exclusion test was used to determine the number of viable cells present in a cell suspension. Following the same conditions, the cells were incubated with nanoparticles for a 24 h at 37 °C and 5% CO_2_. Then, an equal quantity of 0.4% Trypan Blue dye was added to the cell suspension. The mixture was incubated for less than three minutes at room temperature. All cells (blue and clear) were counted on hemocytometer using a light microscope. The results were expressed as a percentage of blue non-viable cells in relation to the total number of cells.

### 2.7. Cellular Uptake and Transfection

Cell uptake of chitosan–heparin and chitosan nanoparticles into ARPE-19 cell line was assessed using Cy5 labeled double stranded VEGF siRNA complexed by the polymers. The transfection of ARPE-19 was performed using chitosan and chitosan–heparin nanoparticles with encapsulated pEGFP-C2.

Firstly the cells were seeded on 96-well optical-bottom plate with Coverglass base (Thermo Scientific^TM^ Nunc^TM^ MicroWell^TM^) with a density of 5 × 10^4^ cells/well in Dulbecco’s Modified Eagle Medium (DMEM-F12) (Biolot, Saint Petersburg, Russia)/10% fetal bovine serum (FBS) (Biowest, South America)/50 IU/mL penicillin/50 µg/mL streptomycin (Biolot, Saint Petersburg, Russia). After 8 h, the medium was removed and 90 µL serum-free DMEM-F12 medium was added to each well. In the cell transfection study, 10 µL of 0.02 mg/mL (200 ng) chitosan or chitosan–heparin nanoparticles with different concentrations of heparin (1:1; 1:2; 1:3 chitosan:heparin mass ratios) and 100 ng of pEGFP-C2 were added to each well (mass ratio chitosan:pDNA = 2:1; N/P = 4.6). To investigate cell uptake, 0.1 nmol of Cy5-siRNA complexed by chitosan or chitosan–heparin at different heparin levels (4:1; 2:1; 1:1 chitosan:heparin mass ratios; mass ratio of chitosan:siRNA was 2:1 equivalent to N/P = 4.6) was added to the wells. The cells were incubated with nanoparticles in serum-free medium for 4 h, then the medium was removed and cells were washed with 1 M NaCl in order to wash out not penetrated particles. After that, 100 µL DMEM-F12 containing 2× FBS and 2× penicillin–streptomycin was added for another 20 h of incubation. Cell uptake and transfection levels obtained with blank Cy5-siRNA and pEGFP-C2 were used as controls.

After 24 h the cells were fixed using exposure of 200 µL 3.7% of formaldehyde–methanol per well for 15 min at 37 °C. Thereafter, the cells were washed three times with PBS. Cell membranes were permeabilized with Permeabilization Buffer (0.2% Triton X-100 in PBS) for 15 min. Staining of the cell nuclei was performed using incubation with Hoechst 33,258 (1 µg/mL) for 30 min according to an established protocol [18]. Then, the cells were washed with PBS for three times, 5 min each time. In order to remove the salts, the cells were washed three times, 2 min each time, with distilled water. Then, the cell membranes were stained by 1× CellMask Green Plasma Membrane Stain (λ_ex_ = 522 nm, λ_em_ = 535 nm; Thermo Fischer Scientific, Paisley, UK), according to the manufacturer’s protocol.

The cell uptake efficiency was determined by analyzing the fluorescence intensity of Cy5-siRNA (λ_ex_ = 650 nm, λ_em_ = 670 nm) using CELENA S Digital Imaging System (Logos Biosystems, GE Healthcare, South Korea) and Cytell Cell Imaging instrument (GE Healthcare, Washington, Issaquah, USA). Transfection efficiency was determined by analyzing the fluorescence intensity of GFP using a microplate reader (λ_ex_ = 488 nm, λ_em_ = 509 nm; (Thermo Scientific Varioskan lux, Vantaa, Finland). Transfection efficiency were calculated as a percentage of GFP fluorescent signal in relation to the average fluorescence of the cells treated with pEGFP-C2 alone.

### 2.8. Gene Silencing of VEGF

The RNA interference potency of 27 bp ds siRNA complexed with chitosan–heparin and directed against VEGF was evaluated in ARPE-19 cells.

ARPE-19 cells were seeded on 24-well plate (Costar TC-treated Multiple Well Plates, Corning, USA) at a density of 2 × 10^5^ cells/well in DMEM-F12 (Biolot, Saint Petersburg, Russia)/10% FBS (Biowest, Nuaille, France)/50 IU/mL penicillin/50 µg/mL streptomycin (Biolot, Saint Petersburg, Russia) overnight prior to siRNA delivery. After that, the medium was removed and 500 µL serum-free DMEM-F12 medium was added to each well. Then, 1 nmol of the siRNA complexed with chitosan–heparin (4:1; 2:1; 1:1 chitosan:heparin mass ratios) was added to each well (mass ratio chitosan:siRNA was 2:1; N/P = 4). To investigate RNA interference in the absence of ds VEGF siRNA, chitosan–heparin complexes with scrambled siControl were added using similar conditions. After 4 h of incubation, the medium was replaced with fresh medium, and the cells were further cultured for 48 h.

The RNA interference against VEGF was evaluated by analyzing the levels of VEGF mRNA with reverse transcription polymerase chain reaction (RT-PCR). Glyceraldehyde-3-phosphate dehydrogenase (GAPDH) mRNA was used as control. The extraction of total RNA was performed using RNA extraction kit from Biosilica according to the manufacturer’s protocol (Biosilica, Novosibirsk, Russia) and the concentration of RNA was determined based on the absorbance at 260 nm. Then, 60 ng of cDNA was synthesized using the MMLV RT kit (Evrogen, Moscow, Russia). Further RT-PCR analysis was conducted using 12 ng of cDNA and the relevant VEGF forward, VEGF reverse, GADPH forward, and GADPH reverse primers. The analyses were carried out using a qPCRmix-HS SYBR (Evrogen, Moscow, Russia) according to the manufacturer’s protocol. The PCR consisted of 45 amplification cycles of 95 °C for 30 s, 55 °C for 30 s, and 72 °C for 45 s.

### 2.9. Statistics

The data were expressed as mean (±SD). Statistical significance of differences (at least 3 measurements for each probe) was determined by one-way analysis of variance (ANOVA) with post hoc test (Bonferroni). Statistical analysis and plotting were performed using PRISM software (GraphPad Prism 5.0, La Jolla, CA, USA). *p* ≤ 0.05 was considered to indicate a statistically significant difference.

## 3. Results and Discussion

### 3.1. Nanoparticle Size, Shape, and Surface Charge

Heparin should be suitable polymeric component in nanomedicines, because it has relatively low molecular weight (12–14 kDa) and it is biodegradable, biocompatible, and non-gelling. However, heparin has been rarely been used for the delivery of gene medicines. To the best of our knowledge, we are the first ones who developed self-assembling chitosan–heparin nanoparticles for cutaneous gene delivery. The ionic crosslinking of the natural polymers is an alternative to covalently cross-linked hydrogels. The electrostatic adhesion between cationic amino groups of chitosan and anionic carboxyl groups of heparin provide a strong interaction in this polyelectrolyte system [15,16,23,24].

Since natural polysaccharides usually have a broad molecular mass distribution and, in some cases, irregular composition, it was important to study the effect of various parameters on particles structure and their stability. Moreover, the size, shape and surface chemistry of nanoparticles can greatly impact cellular uptake and their delivery efficiency in vivo [25]. For determination of particle size, morphology and surface charge various methods were used in this study.

**Figure 1:**
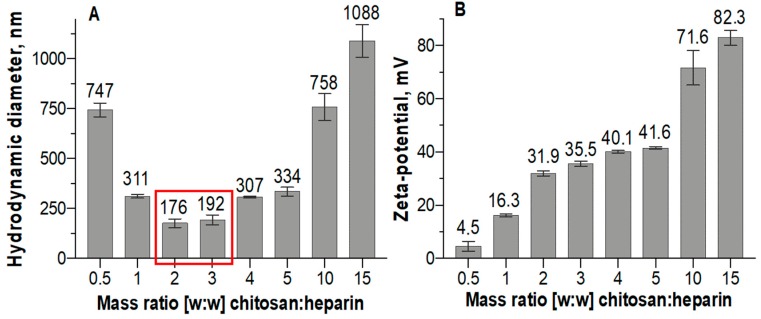
Size (**A**) and charge (zeta-potential) (**B**) of chitosan–heparin nanoparticles prepared with various mass ratios. The smallest sizes are shown with red rectangle. Measurements were performed in 0.01 M PBS, pH 7.4. Mean (±SD), *n* = 3.

Dynamic light scattering showed that chitosan–heparin nanoparticles with mass ratios of 2:1 and 3:1 yielded the smallest particles with mean diameters of 176 nm and 192 nm, respectively (Figure 1A). The agglomeration takes place at isoelectric point (appr. mass ratio of 0.5:1). While an increased amount of chitosan decreases the particle size up to the chitosan/heparin ratio of 3:1, further addition of chitosan increases the particle size (mean size of 1088 nm at 15:1 ratio). The most compact particles with minimum size distribution were obtained at 2:1 chitosan–heparin ratio that can be related to the strongest complexation of two counterparts. The differences in mass ratio represent the relation of polymer weights. The particles formed a stable colloidal solution for at least 72 h.

Higher proportions of chitosan in the particles resulted in higher positive zeta-potentials (Figure 1B). Nevertheless, the surface charge of the nanoparticles increases only slightly at mass ratios from 2:1 to 5:1 and chitosan forms nanoparticles with nearly constant hydrodynamic sizes (Figure 1A) and zeta-potentials (Figure 1B) at these mass ratios. The similar effects have been shown also in hyaluronic acid/chitosan polyelectrolyte complexes [26]. Further increases to chitosan–heparin ratios of 10:1–15:1 forced the nanoparticles to form large aggregates.

We selected chitosan–heparin nanoparticles with a mass ratio of 2:1 for further experiments. These nanoparticles are expected to be colloidally stable, since they minimal size, narrow size distribution and high surface charge. The particles were loaded with negatively charged oligonucleotide dT-dA that resulted in polyplex formation (mass ratio chitosan:oligonucleotide 2:1, N/P = 4.6) between chitosan, heparin and oligonucleotide. The size of the polyplexes was 145 ± 27 nm and mean PDI was 0.21. This PDI demonstrates a narrow size distribution compared to many other nanoparticles [25]. The zeta potential decreased to 24.9 ± 0.6 mV after complexation with the oligonucleotide.

**Figure 2:**
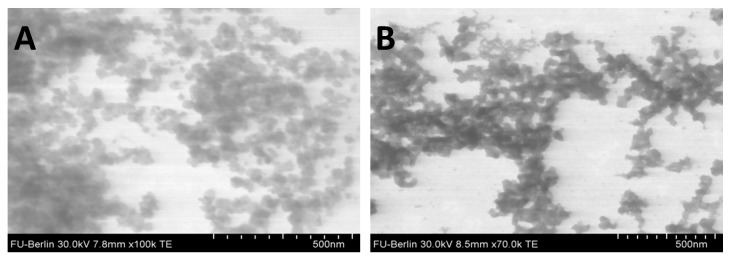
Structure of a chitosan–heparin nanoparticle with mass ratio 2:1 prepared at pH 6.0 (**A**) and pH 8.0 (**B**).

The various chitosan–heparin nanoparticles were studied with scanning-transmission electron microscopy and nanoparticle tracking analysis to get further information about their size and particle structure. Chitosan–heparin nanoparticles were uniformly spherical at both pH 6.0 (Figure 2A) and pH 8.0 (Figure 2B). The particles had a mean diameter of about 50 nm at pH 6.0 and slightly acidic environment caused swelling of individual particles (Figure 2A). In contrast, at pH 8.0 the nanoparticles collapsed and aggregated (Figure 2B), indicating pH-sensitive properties of chitosan–heparin nanoparticles. The different sizes obtained by DLS and STEM are due to the technical differences of both methods and this has been reported earlier [27].

NTA is a method for the determination of a hydrodynamic diameter and size distribution profile of small particles in liquid suspension. NTA tracks the Brownian motion of individual particles or their aggregates at size range of 10–1000 nm and calculates their size taking into account the medium viscosity and particle shape [28].

**Figure 3:**
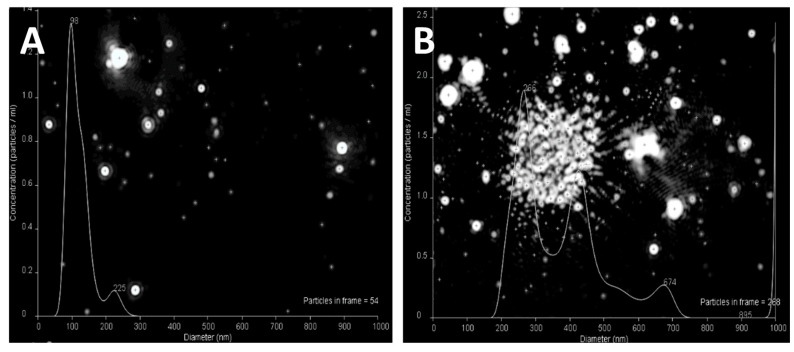
Nanoparticle tracking analysis images of chitosan–heparin nanoparticles with mass ratios of 2:1 (**A**) and 5:1 (**B**) diluted in 0.01 M PBS 7.4.

NTA determination resulted in mean particle size of about 98 nm, smaller diameter than the one obtained with DLS (Figure 1A and Figure 3A). Moreover, small nanoparticles (about 20–30 nm) were clearly visible with the Nanosight system, but harder to capture due to the presence of larger particles that may lead to overexposure when increasing the focus. With increased amounts of chitosan (mass ratio 5:1) (Figure 3B), large conglomerates consisting of small nanoparticles were regularly seen. At increasing chitosan concentration, the nanoparticles formed an ionic network structure in a liquid suspension resulting in size of approximately 1000 nm. These conglomerates are hardly capable of penetrating into the cells or mediating transgene transfection.

### 3.2. Oligonucleotide Entrapment Efficiency

**Figure 4:**
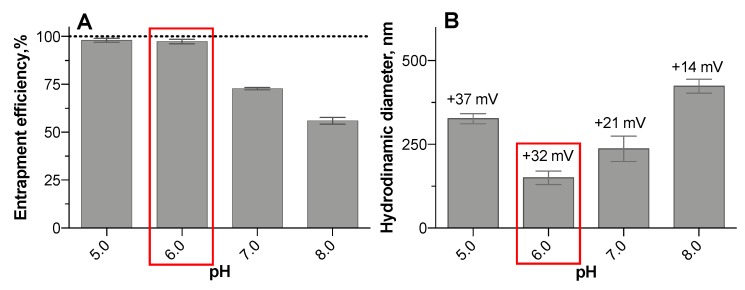
Effect of pH of the medium on entrapment efficiency (**A**), hydrodynamic diameter and zeta-potential (the value above the column) (**B**). Particles with smallest hydrodynamic diameter incubated under pH 6.0 are enclosed in red rectangle. Measurements were performed in various buffer solutions (MES 5.0; MES 6.0; PBS 7.0; PBS 8.0). Mean (±SD), *n* = 3.

In order to deliver genetic materials into the target cells, DNA or siRNA can be bound to polyelectrolyte chitosan–heparin nanoparticles by ionic forces. We used a fluorescently labeled oligonucleotide as an siRNA model. Due to the pH-sensitive nature of chitosan, defined by its pK_a_ 6.0, we examined the impact of the pH value on the entrapment efficiency of oligonucleotide into chitosan–heparin nanoparticles (Figure 4A), as well as their size and zeta-potential (Figure 4B).

Chitosan–heparin complexes encapsulate the oligonucleotide at various degrees, depending on pH of the medium. Almost 100% of labeled oligonucleotide was entrapped at acidic conditions (Figure 4A). The amino groups of chitosan were protonated to the greatest extent in the acidic media resulting in maximal binding efficiency. Thus, the optimal pH for oligonucleotide entrapment has to be around pH 6.0 or below.

The investigations of particle size and zeta-potential at different pH values showed increasing particle size at weakly alkaline conditions due to the deprotonation of amino groups of chitosan that resulted in the aggregation of nanoparticles (Figure 4B). At acidic media with (pH = 5.0) the particle size also increased due to the protonation of amino groups of chitosan followed by the swelling of chitosan–heparin nanoparticles. We assume that protonation/deprotonation processes cause the decreased zeta-potential when pH is shifting from slightly acidic to weakly alkaline media (Figure 4B). Compact and stable nanoparticle complexes were observed at pH 6.0 and below.

Consequently, chitosan–heparin nanoparticles must be prepared at controlled pH conditions to obtain their minimal size.

### 3.3. Oligonucleotide Release

**Figure 5:**
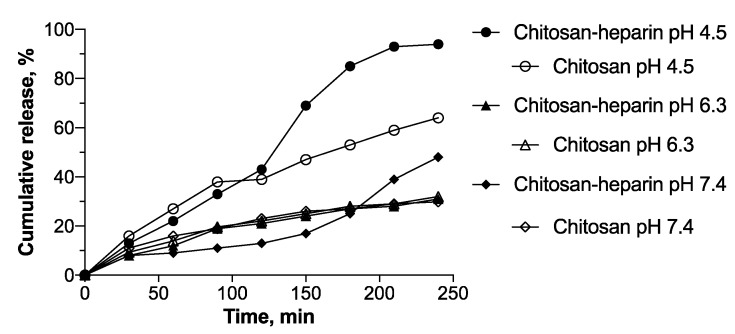
Effect of heparin on oligonucleotide release. Chitosan and chitosan–heparin (mass ratio 2:1) polyplexes with encapsulated Cy3-dT-dA were incubated at pH 4.5 (MES), pH 6.3 (MES), pH 7.4 (PBS) for 240 min.

The impact of added heparin on oligonucleotide release was investigated in media with different pH values, mimicking cytosol and bloodstream (pH 7.5), early endosomes (pH 6.3), or lysosomes (pH 4.5) (Figure 5). Major part of oligonucleotide was released from chitosan and chitosan–heparin nanoparticles in 4 h. The release rate increased especially at pH 4.5, and heparin accelerated oligonucleotide release from the polyplexes at this pH (Figure 5). This might be related to the altered swelling properties of chitosan–heparin nanoparticles. Since heparin is stronger polyanion than oligonucleotide, the displacement of cargo can accelerate release rate. Another reason may be related to breaking of chitosan–heparin nanoparticles into smaller pieces at pH 4.5, which would speed up the release after 2 h of incubation. At endosomal and cytosolic pH, the release was slower in both systems and the curves had similar slopes. At pH 6.3 the release kinetics of both systems were similar (Figure 5). It might be explained by stability factors of nanoparticle colloidal solutions. The increase of pH to 7.4 did not result in a major difference in oligonucleotide release as compared to pH 6.3. Overall chitosan–heparin systems protect the oligonucleotide cargo from degradation and presents relatively fast and complete release at slightly acidic medium, an important factor in facilitation of transfection.

The kinetic patterns were approximated to elucidate the release mechanism [29] (Table 1). The oligonucleotide release from chitosan and chitosan–heparin nanoparticles was best correlated to the Higuchi model (correlation coefficients >0.95). Thus, we assumed that diffusion is an important release mechanism, based on further confirmation by a good correlation with the Baker–Lonsdale model. The release exponent *n* = 1.0–1.2, obtained from a Korsmeyer–Peppas model for chitosan nanoparticles, proved erosion of the chitosan chain to define the cargo release. In contrast, the release exponent *n* = 0.5–0.86 for chitosan–heparin nanoparticles corresponded to non-Fickian diffusion, and, thus proved a combination of both diffusion and erosion to control release rates. The correlation coefficient value *r* was around 0.95 for both release from chitosan and chitosan–heparin, best correlated with zero-order model, i.e., drug release at constant rate. The comparison of correlation coefficient values for zero-order (*r* ≈ 0.95) and first-order (*r* ≈ 0.8) models led us to consider that the release is best correlated with zero-order kinetics. The release coefficients (*k*) obtained by Hixson-Crowell model confirms the process defined by diffusion rather than dissolution of the carrier.

### 3.4. Biocompatibility

After showing the stability of nanoparticles for 48 h in the test medium by DLS, we assessed their cytotoxicity in cultured cells. The hydrodynamic sizes of chitosan and chitosan–heparin nanoparticles (mass ratio 1:1 and 2:1) without cargo after incubation in DMEM during 48 h were not higher than 240 nm, suggesting colloidal stability in the medium. Therefore, we investigated cellular viability with MTT and Trypan Blue assays and primary human keratinocytes. The cells were exposed to chitosan or chitosan–heparin nanoparticles for 24 h and chitosan and heparin bulk materials were used as references.

**Figure 6:**
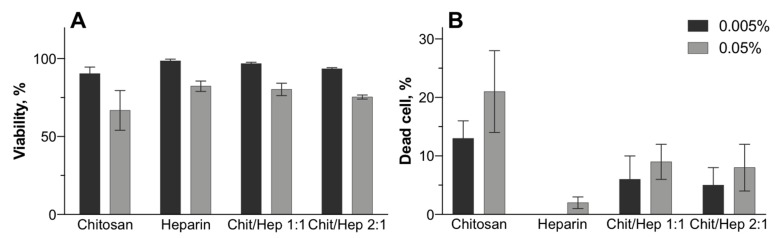
Nanoparticle cytotoxicity in normal human keratinocytes. MTT (**A**) and Trypan Blue Assay (**B**) after 24 h of incubation. Mean (±SD), *n* = 3.

In general, chitosan–heparin nanoparticles induce minor cytotoxicity in human keratinocytes, as the viability declined to 74% in the worst case (Figure 6A). About 18% of cells lost membrane integrity (Figure 6B) for chitosan–heparin nanoparticles at concentration of 0.005%. Nevertheless, higher nanoparticle concentrations only slightly increased the cytotoxicity. This effect remained marginal with minimal viability of 75% and maximum 22% average dead cells. Different chitosan–heparin ratios did not affect cellular toxicity, but the combination of heparin and chitosan improved the safety of the nanoparticles as compared to chitosan alone.

### 3.5. Transfection Efficiency

Cellular DNA-transfection with chitosan based polyplexes has been described previously, and some factors affecting the efficiency (e.g., molecular weight of chitosan, chitosan/DNA ratio, particle size, zeta-potential) have been investigated [7,8,18]. Following endocytic cellular uptake, release of DNA from endo-lysosomal compartment to the cytosol and nucleus was described. It was based on protonation of amino groups in chitosan, consequent particle swelling, and bursting of endosomal membrane [7]. There are a few reports about inclusion of polyanions into chitosan-based polyplexes, namely hyaluronic acid [26] or sodium alginate [30]. The transfection of epithelial cells with those polyplexes with pDNA or siRNA resulted in better cell penetration and release of the cargo as compared to chitosan alone [26]. However, the effects of heparin in the chitosan-based polyplexes have not been studied earlier.

**Figure 7:**
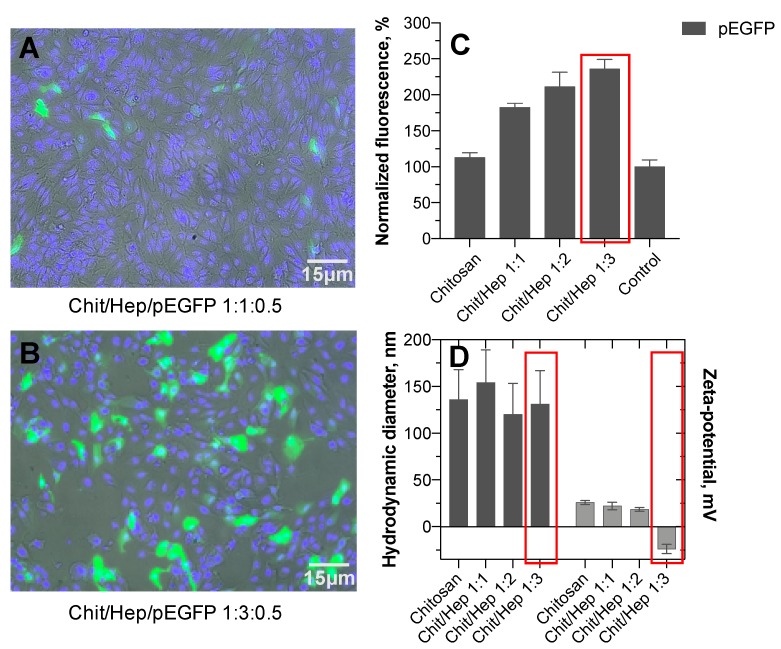
Transfection of ARPE-19 cells by chitosan–heparin-pEGFP polyplexes at mass ratio 1:1:0.5 (**A**) and 1:3:0.5 (**B**). Normalized fluorescence of ARPE-19 cells after transfection with pEGFP chitosan–heparin polyplexes (**C**). Transfected cells using pEGFP alone were used as negative control and the transfection data were normalized to this negative control. Hydrodynamic diameter (DLS) and surface zeta-potential of polyplexes (**D**). The particle size (black bars) and zeta potential (grey bars) of the polyplexes. The formulation with the best transfection efficiency is marked with red rectangles. Mean (±SD), *n* = 3.

Human retinal epithelial cell line was transfected with the reporter gene that encodes green fluorescence protein using chitosan and chitosan–heparin nanoparticles. The fluorescence signal was used as an indicator of transfection efficiency (Figure 7).

The data demonstrates that the cells were transfected at much higher efficiency with chitosan–heparin–pEGFP polyplexes as compared to the control transfection with pEGFP-C2 alone. Furthermore, chitosan–heparin-based polyplexes exhibited higher transfection efficiency than chitosan nanoparticles with pEGFP.

Transfection with chitosan and chitosan–heparin based nanoparticles depends on the cellular endocytosis of polyplexes. DNA release from the polyplexes is believed to proceed through proton sponge effect due to the swelling of chitosan–heparin complexes in the endo-lysosomes [31]. Chitosan is known to be a strong polycation with high charge density. Thus, the inclusion of heparin, (polyanionic glycosaminoglycan) on the nanoparticles was proposed to enhance DNA release and transfection of cells due to interactions between heparin and chitosan, which may lead to DNA displacement and its release to the cytoplasm.

Polyplexes were also characterized in terms of particle size, polydispersity index and zeta-potential. Excess of heparin on the nanoparticle surface at a chitosan:heparin mass ratio of 1:3 resulted in negative surface charge (zeta-potential −22 ± 4 mV). However, there were no significant size differences among polyplexes at different chitosan:heparin mass ratios (Figure 7D). Hence, the polyplexes with negative zeta potentials were capable to bind DNA and transfer it into the cells.

The lowest rate of transfection was obtained with pEGFP-C2 alone. Chitosan–pEGFP formulation increased the tranfection levels only by 17% (Figure 7C). In contrast, GFP expression after chitosan/heparin polyplex transfection was almost two times higher than that of chitosan polyplexes (Figure 7C). In addition, significant differences were found among polyplexes with different chitosan/heparin mass ratios, with chitosan/heparin polyplexes at a mass ratio 1:3 showing the highest transfection levels (Figure 7C).

### 3.6. Gene Silencing of VEGF

Vascular endothelial growth factor is an endothelial cell-specific mitogen and an angiogenesis inducer in vivo. Its activity has been linked to tumor growth and formation of metastases [32]. VEGF has been implicated in the disruption of retinal pigment epithelium barrier function and accumulation of subretinal fluid from the leaky neo-vessels. Therefore, it is important to find a powerful technologies for VEGF inhibition [33].

RNA interference technologies are currently widely used in functional genomic studies [34,35,36]. Also, small interfering RNA is being developed as therapeutics against cancers and other indications [37]. However, the drug delivery systems for siRNA have not been fully developed for in vivo use. We studied the use of chitosan–heparin nanoparticles and the role of heparin amount in these polyplexes for the delivery of anti-VEGF siRNA into ARPE-19 cells.

**Figure 8:**
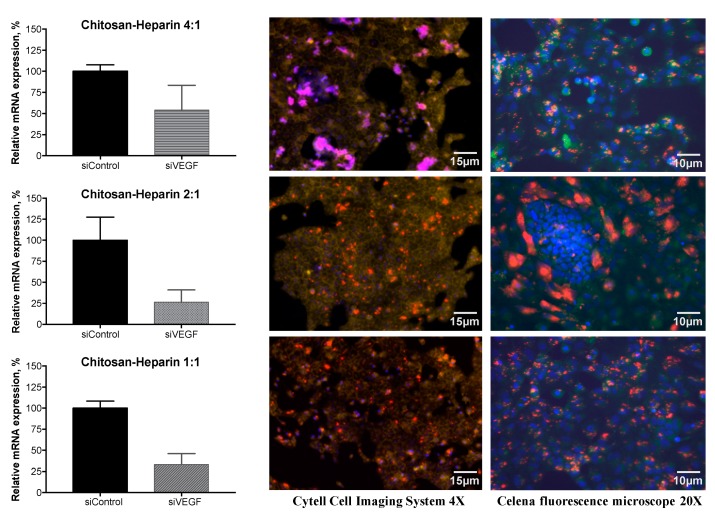
RNA interference by anti-VEGF small interfering RNA that was delivered in chitosan–heparin nanoparticles at different mass ratios (1:1; 2:1; 4:1) in the ARPE-19 cell line. LEFT. Relative VEGF mRNA expression data from RT-PCR assays are shown (left panel). GADPH mRNA was measured as an intrinsic control. Mean (±SD), *n* = 3. RIGHT. Fluorescence microscopy images of ARPE-19 cells that were transfected with Cy5-dsRNA (red color) complexed by chitosan–heparin at different mass ratios (1:1; 2:1; 4:1). The cell nuclei were stained by Hoechst 33,258 (blue color) and the plasma membranes were stained using CellMask Green Plasma Membrane Stain (yellow and green color).

We investigated the RNA interference efficiency of anti-VEGF siRNA [21] in ARPE-19 cells that express VEGF constitutively. The VEGF mRNA expression in the cells was analyzed by RT-PCR. Chitosan–heparin polyplexes at mass ratios 1:1 and 2:1 demonstrated two times stronger inhibitory effects than chitosan–heparin polyplexes at a 4:1 mass ratio (Figure 8).

Fluorescence microscopy images of ARPE-19 cells treated with Cy5-dsRNA–chitosan–heparin polyplexes showed effective cell penetration of Cy5-labeled dsRNA. In the case of 1:1 and 2:1 chitosan–heparin mass ratios, most siRNAs were located in the cytoplasm (Figure 8). At a 4:1 mass ratio, chitosan–heparin particles have larger particle size due to the aggregate formation. Hence, the particle penetration into the cells was partially limited (Figure 8).

Overall, addition of heparin to chitosan-based polyplexes enhanced siRNA and DNA release and cytosolic delivery, thereby improving gene silencing and transgene expression in the cells.

## 4. Conclusions

In present work, biocompatible nanoparticles based on chitosan–heparin complexes were developed. The particles were prepared in mild conditions using a drop-wise addition method. The model oligonucleotide was associated with cationic chitosan–heparin complexes via ionic interactions that led to the formation of chitosan–heparin-oligonucleotide polyplexes. The pH-sensitive behavior of nanoparticles was demonstrated. DLS, NTA, and STEM methods informed that the mean particle sizes of the particles were 100–200 nm depending on the heparin amount. The increasing chitosan concentrations in the polyplexes led to the particle aggregation and increased particle size (400–1000 nm). The in vitro release studies and mathematical modelling of the release kinetics demonstrated increased release rate of oligonucleotide at slightly acidic conditions (pH 4.5) due to swelling and diffusion of cargo. In vitro release from chitosan–heparin polyplexes was faster than from chitosan polyplexes, possibly due to the effect of heparin as competitive polyanion.

The cell viability tests demonstrated lack of cytotoxicity for chitosan–heparin. Addition of heparin to the polyplexes did not affect cellular uptake, but increased the DNA-transfection efficiency in the ARPE cells. Likewise, VEGF silencing in the ARPE-19 cells was enhanced when heparin was used in the chitosan polyplexes. These improved effects were facilitated by the enhanced release of siRNA in the cells. This approach could be used to increase the rate of cargo release from other nanocarriers. The chitosan–heparin nanocarriers also have potential for the delivery of genetic drugs into epithelial tissues because they may open the epithelial tight junctions and they have mucoadhesive properties.

## Figures and Tables

**Table 1:** Release kinetics of oligonucleotides from chitosan or chitosan–heparin nanoparticles, with *r* as correlation coefficient value, *k* as the release constant, and *n* as the diffusion or release exponent.

Nanoparticle	pH	Model					
		Zero Order	First Order	Higuchi	Hixson-Crowell	Korsmeyer–Peppas	Baker–Lonsdale
Chitosan	4.5	*r* = 0.9498	*r* = 0.8150	*r* = 0.9445	*r* = 0.8741	*r* = 0.9394	*r* = 0.9430
		*k* = 13.48	*k* = −0.51	*k* = 28.52	*k* = −0.84	*n* = 1.26	*k* = 8.41
	6.3	*r* = 0.9537	*r* = 0.7897	*r* = 0.9704	*r* = 0.8662	*r* = 0.9688	*r* = 0.9583
		*k* = 16.13	*k* = −0.41	*k* = 34.47	*k* = −0.78	*n* = 1.04	*k* = 14.61
	7.4	*r* = 0.9811	*r* = 0.9037	*r* = 0.9400	*r* = 0.9478	*r* = 0.9489	*r* = 0.8687
		*k* = 33.80	*k* = −0.47	*k* = 70.43	*k* = −1.09	*n* = 1.12	*k* = 78.14
Chitosan–heparin	4.5	*r* = 0.9644	*r* = 0.7834	*r* = 0.9943	*r* = 0.8651	*r* = 0.9795	*r* = 0.9841
		*k* = 6.52	*k* = −0.33	*k* = 14.01	*k* = −0.57	*n* = 0.86	*k* = 2.42
	6.3	*r* = 0.9666	*r* = 0.8193	*r* = 0.9535	*r* = 0.8517	*r* = 0.9506	*r* = 0.9639
		*k* = 6.80	*k* = −0.24	*k* = 19.06	*k* = −0.45	*n* = 0.59	*k* = 5.91
	7.4	*r* = 0.9377	*r* = 0.7855	*r* = 0.9839	*r* = 0.8483	*r* = 0.9768	*r* = 0.9836
		*k* = 14.40	*k* = −0.26	*k* = 31.32	*k* = −0.56	*n* = 0.66	*k* = 16.87
